# Rate-Gyro-Integral Constraint for Ambiguity Resolution in GNSS Attitude Determination Applications

**DOI:** 10.3390/s130607979

**Published:** 2013-06-21

**Authors:** Jiancheng Zhu, Tao Li, Jinling Wang, Xiaoping Hu, Meiping Wu

**Affiliations:** 1 Department of Automatic Control, College of Mechatronics and Automation, National University of Defense Technology, Deya Street 109, Changsha 410073, Hunan, China; E-Mails: Xphu@nudt.edu.cn (X.H.); meipingwu@263.net (M.W.); 2 School of Surveying and Geospatial Engineering, University of New South Wales, Sydney, NSW 2052, Australia; E-Mail: Jinling.Wang@unsw.edu.au

**Keywords:** ambiguity search space, GNSS attitude determination, rate-gyro-integral constraint, land vehicle application

## Abstract

In the field of Global Navigation Satellite System (GNSS) attitude determination, the constraints usually play a critical role in resolving the unknown ambiguities quickly and correctly. Many constraints such as the baseline length, the geometry of multi-baselines and the horizontal attitude angles have been used extensively to improve the performance of ambiguity resolution. In the GNSS/Inertial Navigation System (INS) integrated attitude determination systems using low grade Inertial Measurement Unit (IMU), the initial heading parameters of the vehicle are usually worked out by the GNSS subsystem instead of by the IMU sensors independently. However, when a rotation occurs, the angle at which vehicle has turned within a short time span can be measured accurately by the IMU. This measurement will be treated as a constraint, namely the rate-gyro-integral constraint, which can aid the GNSS ambiguity resolution. We will use this constraint to filter the candidates in the ambiguity search stage. The ambiguity search space shrinks significantly with this constraint imposed during the rotation, thus it is helpful to speeding up the initialization of attitude parameters under dynamic circumstances. This paper will only study the applications of this new constraint to land vehicles. The impacts of measurement errors on the effect of this new constraint will be assessed for different grades of IMU and current average precision level of GNSS receivers. Simulations and experiments in urban areas have demonstrated the validity and efficacy of the new constraint in aiding GNSS attitude determinations.

## 1. Introduction

Ambiguity resolution is a core technique in GNSS relative positioning and attitude determination. There is no essential difference between the two applications in terms of this technique. Unlike the relative positioning, however, the distances between antennas are constant and short in attitude determination, generally ranging from a few meters to dozens of meters. Therefore, more constraints exist in attitude determination and the double-difference (DD) carrier phase measurement is more precise than relative positioning. These constraints have become very important for ambiguity resolution in attitude determination.

There exist many constraints applied to ambiguity resolution. The baseline length is a readily available and widely used constraint. The ambiguity search space can be reduced from 3D to 2D by using this constraint [1]. The elevations measured by a tilt meter were used for aiding ambiguity resolution in a single baseline orientation determination system [2]. The correct ambiguity combination can be fast fixed with the two constraints under static condition [3]. Otherwise, both the baseline length and trigonometric function constraints can be used to shrink an expanded searching ellipsoidal space to a proper size, which maintains the true integer ambiguity [4]. In the multi-baselines cases, the ambiguity search space can be further reduced to 1-D by geometrical constraint [5]. Then a recursive ambiguity search algorithm using both the length and geometrical constraints was proposed [6]. The latest contribution, namely the Weighted Constrained Least-squares AMBiguity De-correlation Adjustment (WC-LAMBDA) method, was proposed based on integrating the nonlinear baseline constraint into the ambiguity objective function of LAMBDA method. This method dose full justice to the given information of the baseline length [7]. IMU measurements usually benefit the ambiguity resolution in GNSS/INS attitude determination system. Outlier in the up-component of baseline solution can be detected more readily by exploiting the pitch axis angular rate measured by a Fiber-Optical-Gyroscope (FOG). A coarse ambiguity resolution was estimated by using the FOG data and GNSS observations, and the final ambiguity resolution was obtained from the DD carrier phase observation equations together with aid of the coarse resolution [8]. Gyroscope measurements were demonstrated to be efficient in improving the robustness of ambiguity resolution under real-time dynamic conditions [9].

Platform rotations can produce more information used in ambiguity resolution. This idea was proposed by [10] originally. It utilizes both baseline length constraint and differential vector estimations for ambiguity resolution. Following this idea, [11] conducted a comprehensive research on the GNSS attitude determination technique. And inspired by the idea of antenna swap method, [12] proposed an ambiguity resolution method based on rotational motions. However, the model of this method is too idealistic to implement. On the basis of Tu's research, another method based on two-degree-of-freedom rotational motions was carried out by [13]. But the validity of ambiguity resolution is disturbed by the complex rotating mechanism and inaccurate rotation angle measurements in practice. The characteristics of the single-difference carrier phase variations between adjacent epochs were studied by [14], under the rotation conditions. They designed a single-difference ambiguity fixing method under the condition that the rotating axis was fixed. The influence of actual measurement errors on ambiguity resolution was discussed as well. In the recent years, the platform motions were utilized to enhance the anti-cycle-slip capability of ambiguity resolution, and the methods basing on this fact were demonstrated to be especially efficient in land vehicle attitude determination [15,16].

In this paper, a new constraint is proposed for the ambiguity resolution in GNSS attitude determination applications. For land vehicle applications, the baseline approximately lies in the plane of local level during a rotational motion [17]. Thus the relative rotation angle measurements provided by IMU are used to aid ambiguity resolution. As shown below, this constraint does not require high accuracies of IMU sensors, especially for the accuracy of gyroscope.

The rest part of this paper is organized as follows: Section 2 summarizes the basic principle and mathematic model for the rate-gyro-integral constraint. Section 3 presents a specific implementation method and its geometric analysis. Section 4 discusses on the error factors of the proposed implementation method. Section 5 gives some simulation results. Section 6 shows the results of processing actual field data. Section 7 concludes this paper and gives some suggests for future research.

## 2. Basic Principle and Mathematical Model

The rotational motions of vehicle can be measured by gyroscopes that are part of the IMU. In this section, our work is to find the proper mathematical models for explicating how this measurement improves the efficiency of ambiguity search.

### 2.1. Inertial Baseline Vector Solution

It is assumed that an IMU is attached to the vehicle with two GNSS antennas. The b-frame has its origin at the IMU reference point. The longitudinal axis of the vehicle is the X axis of the b-frame and the transvers axis of the vehicle is the Y axis of the b-frame. The Z axis obeys right-handed rule and points downwards. The baseline vector *a⃑*^(*b*)^ is the 3D vector between the carrier phase centers of the two antennas, with three known constants being its coordinates in the b-frame. The origin of the n-frame (North, East, Down) is consistent with that of the b-frame. If $C_{b}^{n}$ denotes the transformation matrix from the b-frame to the n-frame, which is also the pattern to represent vehicle attitude in our case, then ${\vec{a}}^{\left(n\right)}(t_{0})=C_{b}^{n}(t_{0}){\vec{a}}^{\left(b\right)}$ is obtained, where *a⃑*^(*n*)^ is the baseline vector in the n-frame and *t*_0_ is the starting time of rotation.

Updating $C_{b}^{n}$ is primarily implemented through integrating the outputs of the IMU. Starting from $C_{b}^{n}(t_{o})$, the strapdown mechanization can independently derive the attitude solution at each update moment of the IMU outputs. Without regard to the measurement errors, the so-called inertial baseline vector solution is given by ${\vec{a}}^{\left(n\right)}(t_{k})=C_{b}^{n}(t_{k}){\vec{a}}^{\left(b\right)},t_{k}>t_{0},t_{k}$ denotes any update moment after rotation begins. Then the inner product of *a⃑*^(*n*)^(*t*_0_) and *a⃑*^(*n*)^ (*t_k_*) can be written as:

(1) $$S_{k}={\vec{a}}^{\left(n\right)}{\left(t_{0}\right)}^{T}\cdot {\vec{a}}^{\left(n\right)}(t_{k})={\vec{a}}^{(b)T}C_{b}^{n}{\left(t_{0}\right)}^{T}\cdot C_{b}^{n}(t_{0})A_{k}{\vec{a}}^{\left(b\right)}={\vec{a}}^{(b)T}A_{k}{\vec{a}}^{\left(b\right)}$$

where *A_k_* is the inertial attitude update matrix, it mainly depends on the integral of gyroscope outputs in the interval of [*t*_0_, *t_k_*], thus *S_k_* is a function of *A_k_*. Equation (1) shows that $C_{b}^{n}$ (*t*_0_) has no effect on *S_k_*. By applying the definition of inner product, we can write an equivalent form of Equation (1) as:

(2) $$\cos\alpha_{k}=S_{k}/\left|{\vec{a}}^{\left(n\right)}(t_{0})\left|\cdot \right|{\vec{a}}^{\left(n\right)}(t_{k})\right|={\vec{e}}^{(b)T}A_{k}{\vec{e}}^{\left(b\right)}$$

where *e⃑*^(*b*)^ is the unit vector of *a⃑*^(*b*)^ and *a_k_* represents the angle between *a⃑*^(*n*)^ (*t*_0_) and *a⃑*^(*n*)^ (*t_k_*).

### 2.2. GNSS Baseline Vector Solution

The GNSS DD carrier phase observation equations are formulated as follows [12]:

(3) $$\begin{array}{l} G{\tilde{\vec{a}}}^{\left(n\right)}=\tilde{K}\\ G={\left[G_{2}-G_{1}\;\cdots \;G_{n+1}-G_{1}\right]}^{T}\\ \tilde{K}=\lambda {\left[{\tilde{k}}_{1}\;\cdots \;{\tilde{k}}_{n}\right]}^{T},{\tilde{k}}_{1}=\nabla \Delta \phi^{i}+\nabla \Delta N^{i}+\nabla \Delta b_{\phi }^{i} \end{array}$$

where *G_i_* is the unit vector in the line of sight (LOS) and heading to satellite i. ∇Δ*ϕ^i^* and ∇Δ*N^i^* denote the DD carrier phase observation and integer ambiguity, respectively. $\nabla \Delta b_{\phi }^{i}$ denotes the observable noise.

Four satellites with minimum Geometry Dilution of Precision (GDOP) value are selected from all visible satellites, and the satellite with the largest elevation is chosen as the “reference” satellite, the other three satellites are recognized as the “master satellites”. Thus there are three independent DD carrier phase measurement equations in Equation (3). For the altitude of GPS satellite is about 20,200 km above the sea level and the displacement of land vehicle is limited within a short time, *G* can be approximately viewed as a constant matrix. If the integer ambiguity vector is known, Equation (3) will be solved without observable noise and the so-called GNSS baseline vector solution will be obtained:

(4) $$\begin{array}{l} {\vec{a}}^{(n)'}={\left(G^{T}G\right)}^{-1}G^{T}K\\ K=\lambda {\left[k_{1}\;k_{2}\;k_{3}\right]}^{T},k_{i}=\nabla \Delta \phi^{i}+\nabla \Delta N^{i} \end{array}$$

where *G* is nonsingular. In order not to be mistaken for the inertial baseline vector solution, this GNSS baseline vector solution is denoted as *a⃑*^(*n*)′^. Thus, the inner product of the GNSS baseline vector solutions at *t*_0_ and *t_k_* can be described as:

(5) $$S_{k}^{'}={\vec{a}}^{\left(n\right)}{\left(t_{0}\right)}^{'T}\cdot {\vec{a}}^{\left(n\right)}(t_{k})'=K{\left(t_{0}\right)}^{T}[G^{-1T}G^{-1}]K(t_{k}).$$

Similarly, the angle between *a⃑*^(*n*)^ (*t*_0_)′ and *a⃑*^(*n*)^ (*t_k_*)′ is denoted as $\alpha_{k}^{'}$.

Hence, if the true integer ambiguity combination, which is denoted as ∇Δ*N⃑*, is substituted into Equation (4), the correct GNSS baseline vector solution will be obtained. However, if the known integer ambiguity vector in Equation (4) is incorrect and the corresponding bias is denoted as *δN⃑_m_*, this will deduce incorrect baseline solutions and inner products. With Equations (4) and (5), we obtain:

(6) $$\begin{array}{l} S_{k\;m}^{'}={\vec{a}}^{\left(n\right)}{{\left(t_{0}\right)}_{m}^{'}}^{T}\cdot {\vec{a}}^{\left(n\right)}{\left(t_{k}\right)}_{m}^{'}\\ ={\left[K(t_{0})+\delta {\vec{N}}_{m}\right]}^{T}[G^{-1T}G^{-1}][K(t_{k})+\delta {\vec{N}}_{m}]=S_{k}^{'}+\delta S_{k}^{'}\\ \delta S_{k}^{'}={\left(G^{-1}\delta {\vec{N}}_{m}\right)}^{T}G^{-1}K(t_{k})+{\left(G^{-1}K(t_{0})\right)}^{T}G^{-1}\delta {\vec{N}}_{m}+{\left|G^{-1}\delta {\vec{N}}_{m}\right|}^{2} \end{array}$$

where *a⃑*^(*n*)^ (*t*_0_)′ *_m_* and *a⃑*^(*n*)^(*t_k_*)′ *_m_* denote the incorrect baseline solutions. $\delta S_{k}^{'}$ and $\alpha_{k\;m}^{'}$ denote the bias of the inner product and the angle between *a⃑*^(*n*)^ (*t*_0_)′*_m_* and *a⃑*^(*n*)^(*t_k_*)′*_m_*, respectively.

### 2.3. Rate-Gyro-Integral Constraint

**Figure 1.**
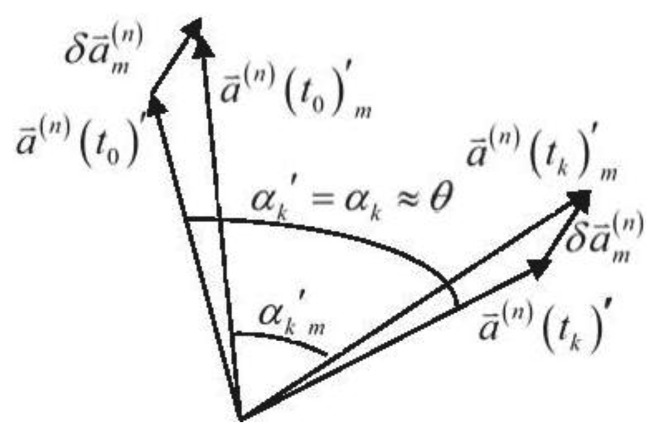
Geometric depiction of the rate-gyro-integral constraint.

For land vehicles, the baseline approximately lies in the local level. Thus, $\alpha_{k}^{'}$ is very close to the angle, which is denoted as *θ*, that vehicle has turned at around an “equivalent rotation axis” from *t*_0_ to *t_k_*. For the precision of integral of gyroscope outputs is high enough within a short time span, *α_k_* is always close enough to *θ* as well. This can be utilized as a constraint, namely the rate-gyro-integral constraint, for filtering the ambiguity candidates in the search space, e.g., one of them is denoted as ∇Δ*N⃑_m_*, deduces a $\alpha_{k,m}^{'}$ that is far from *α_k_*. Figure 1 depicts this new constraint:

In Figure 1, $\delta {\vec{a}}_{m}^{\left(n\right)}$ is a constant vector. It will directly result in the difference between $\alpha_{k}^{'}$ and $\alpha_{k\;m}^{'}$ once the rotational motion starts.

## 3. Implementation and Geometric Analysis

An implementation method for the rate-gyro-integral constraint is proposed in this section. By comparing the testing objectives with a properly selected threshold, the unacceptable testing objectives can be found out and the corresponding candidates are filtered out from the ambiguity search space.

### 3.1. Implementation Method

The mathematical description of a rotational motion usually consists of a rotation axis and a rotation angle. The projections of *a⃑*^(*n*)^(*t*_0_)′ and *a⃑*^(*n*)^(*t_k_*)′ on the rotation plane, are denoted as *a⃑*^(*n*)^ (*t*_o_)′ and *a⃑*^(*n*)^ (*t_k_*)′, respectively. Herein, it should be noted that $\alpha_{k}^{'}$ expresses the angle between *a⃑*^(*n*)^ (*t*_o_)′ and *a⃑*^(*n*)^ (*t_k_*)′. Similarly, the projections of *a⃑*^(*n*)^(*t*_0_)′*_m_* and *a⃑*^(*n*)^(*t_k_*)′*_m_* are denoted as *a⃑*^(*n*)^(*t*_0_)′*_m_* and *a⃑*^(*n*)^(*t_k_*)′*_m_*, respectively, and the angle between them is expressed as $\alpha_{k\;m}^{'}$.

Assuming that the rotation angle can be measured by the IMU sensors, thus it is easy to verify that $\theta =\alpha_{k}^{\prime }=\alpha_{k}$, in which *θ* and *α_k_* denote the rotation angle and its IMU measurement, respectively. At this point, the testing objective, which is expressed as Δ*α_k,m_* = *α_k,m_*′ −*θ*, is defined. Then a threshold denoted as |Δ*α*|_threshold_ is selected properly for the implementation method.

**Figure 2.**
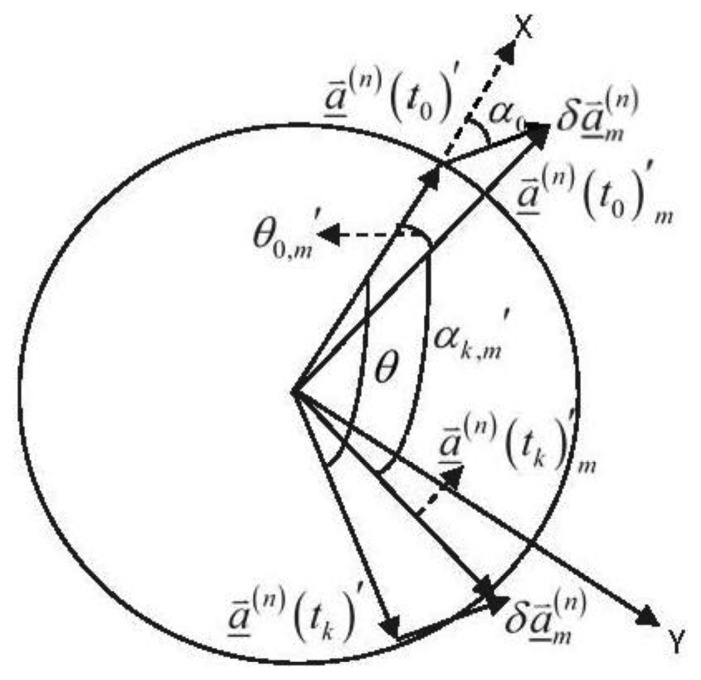
Projection vectors and angles on the rotation plane.

At the ambiguity search stage, if a candidate ∇Δ*N⃑_m_* deduces a Δ*α_k,m_* that satisfies |*Δ_k,m_*|>|Δ*α*|_threshold_, thus the testing objective is verified to be unacceptable and ∇Δ*N⃑_m_* will be filtered out from the search space. Figure 2 delineates the projection vectors and the angles on the rotation plane.

Assuming that the baseline vector rotates a whole round, thus, in Figure 2, *θ* and *α*_*k*,*m*_′ lie inside the interval of [0°,360°], and the other notations will be illustrated later.

### 3.2. Geometric Analysis

In Figure 2, the baseline vector lies in the rotation plane all the time and turns 360° around a fixed rotation axis clockwise. The baseline length is considered as a constant *L.* The rotational angular rate is assumed to be a constant value *360*/*m* (°/s)(*m* ≥ *k*, *m*, *k* ∈ ℤ^+^). A planar Cartesian coordinate, namely the p-frame, is defined on the rotation plane. The X-axis of the p-frame is consistent with the baseline vector at *t*_0_, and the Y-axis of the p-frame vertically points to the right of X-axis. *θ* ranges from 0° to 360°. The difference between projections of the true baseline vector and an incorrect baseline vector solution is denoted as $\delta {\underline{\vec{a}}}_{m}^{\left(n\right)}$. For an incorrect ambiguity candidate deducing it, together with Equation (4), it is verified that $\delta {\underline{\vec{a}}}_{m}^{\left(n\right)}$ is a constant vector and its length is denoted as *δL.* By some simple algebraic operations, the analytic formula for calculating the testing objective is given by (the detailed derivation can be found in the Appendix):

(7) $$\Delta \alpha_{k,m}=\{\begin{array}{c} \tan2^{-1}\left(\frac{L\sin\theta +\delta L\sin\alpha_{0}}{L\cos\theta +\delta L\cos\alpha_{0}}\right)-\tan2^{-1}\left(\frac{\delta L\sin\alpha_{0}}{L+\delta L\cos\alpha_{0}}\right)-\theta \\ ,\theta \in [0,2\pi -\sin^{-1}\left(\frac{\delta L\sin\alpha_{0}}{L}\right))\\ \tan2^{-1}\left(\frac{L\sin\theta +\delta L\sin\alpha_{0}}{L\cos\theta +\delta L\cos\alpha_{0}}\right)-\tan2^{-1}\left(\frac{\delta L\sin\alpha_{0}}{L+\delta L\cos\alpha_{0}}\right)-\theta +2\pi \\ ,\theta \in [2\pi -\sin^{-1}\left(\frac{\delta L\sin\alpha_{0}}{L}\right),2\pi ] \end{array}$$

where *α_0_* represents the angle between $\delta {\underline{\vec{a}}}_{m}^{\left(n\right)}$ and the X-axis of the p-frame; *θ*_0_,*_m_*′ denotes the angle between *a⃑*^(*n*)^(*t*_0_)′*_m_* and the X-axis of the p-frame; tan 2^−1^ (•) is defined as follows:

(8) $$\tan2^{-1}\left(\frac{y}{x}\right)=\{\begin{array}{l} \tan^{-1}(y/x),x>0,y>0\\ \pi /2,x=0,y>0\\ \pi +\tan^{-1}(y/x),x<0\\ 3\pi /2,x=0,y<0\\ 2\pi +\tan^{-1}(y/x),x>0,y<0\\ 0,x>0,y=0 \end{array}$$

Taking the derivative of the right side of Equation (7) with *θ* and then making the result equal to zero, we can obtain:

(9) $$\cos(\theta -\alpha_{0})=-\frac{\delta L}{L}.$$

During the whole rotation procedure, there are two *θ* s satisfy Equation (9). Substituting them into Equation (7), two peak values of Δ*α_k,m_* will be attained at *t_k_*_1_ and *t_k_*_2_ respectively. Then, just denote the larger one of the abstract values of the two peak values as:

(10) $$\Delta \alpha_{m}^{\text{MAX}}=\max\left\{\left|\Delta \alpha_{k1,m}\right|,\left|\Delta \alpha_{k2,m}\right|\right\}.$$

Finally, the explicit expression of $\Delta \alpha_{m}^{\text{MAX}}$ can be given by:

(11) $$\begin{array}{ll} \Delta \alpha_{m}^{MAX}= & \left|\cos^{-1}(\delta L/L)-\pi /2-\theta_{0,m}^{'}\right|\\ \theta_{0,m}^{'}= & \tan2^{-1}\left(\frac{\sin\alpha_{0}(\delta L/L)}{1+\cos\alpha_{0}(\delta L/L)}\right). \end{array}$$

According to Equation (11), it is a naive thought that $\Delta \alpha_{m}^{\text{MAX}}$ represent the fullest potential of the implementation method to identify the incorrect candidate ∇Δ*N⃑_m_*.

In brief, there are two important elements of the implementation method for the rate-gyro-integral constraint. One is to generate a set of testing objectives for each candidate, the other is to select a proper threshold. For the former, it is necessary to investigate two aspects, the distribution range and density of the testing objectives on the rotation process. For the latter, the success rate and shrinking efficiency are analyzed under different conditions of measurement scenarios and threshold settings.

## 4. Error Analysis

Contributors to the inaccuracy of testing objective involve the IMU measurement errors, especially those associated with angle rate, GNSS carrier phase measurement errors and the actual rotational axis offsets.

The angle rate measurement errors are largely responsible for the inaccuracy of testing objective by IMU measurement effects. In the strapdown mechanization, the measurement model of angle rate with respect to the n-frame can be expressed as [18]:

(12) $${\tilde{\vec{\omega }}}_{nb}^{b}={\vec{\omega }}_{nb}^{b}+\delta {\vec{\omega }}_{nb}^{b},\;\delta {\vec{\omega }}_{nb}^{b}=\delta {\vec{\omega }}_{ib}^{b}-\delta {\vec{\omega }}_{ie}^{b}-\delta {\vec{\omega }}_{en}^{b}.$$

Note that the second formula in Equation (12) is the error model, in which the error term associated with gyroscope is denoted as $\delta {\vec{\omega }}_{ib}^{b}$, and one of its routine options is given by [18]:

(13) $$\delta {\vec{\omega }}_{ib}^{b}=\vec{b}+\vec{w}$$

where *b⃑* and *w⃑* are the bias and noise of gyroscope measurement, respectively.

Since the attitude of vehicle is unknown, $\delta {\vec{\omega }}_{ie}^{b}$ can be simplified as $-{\vec{\omega }}_{ie}^{b}$. Similarly, if translational motion of vehicle occurs, $\delta {\vec{\omega }}_{en}^{b}$ will be simplified as $-{\vec{\omega }}_{en}^{b}$. Such that from *t*_0_to *t_k_*, a rotational angle measurement, denoted as *θ͂* , can be derived by the strapdown mechanization. The measurement errors contained in *θ˜* are primarily driven by $\delta {\vec{\omega }}_{nb}^{b}$. Thus the effect induced by IMU measurement errors on the testing objective can be expressed as:

(14) $$\delta \theta =\tilde{\theta }-\theta =\left\|\int_{t_{0}}^{t_{k}}{\tilde{\vec{\omega }}}_{nb}^{b}(\tau )d\tau \right\|-\left\|\int_{t_{0}}^{t_{k}}{\vec{\omega }}_{nb}^{b}(\tau )d\tau \right\|$$

where ‖•‖ denotes the norm function. By applying the inequality law, an upper bound of *δθ* is obtained:

(15) $$\left|\delta \theta \right|\leq \left\|\delta \vec{\theta }\right\|$$

with:

(16) $$\delta \vec{\theta }=\int_{t_{0}}^{t_{k}}\delta {\vec{\omega }}_{nb}^{b}(\tau )d\tau .$$

For land vehicle rotational motions, the unit vector of local gravitational vector, which has three constant coordinates in the n-frame and is denoted as *x⃑*^(*n*)^′ = [0 0 1]*^T^* , can be treated as an observation of actual rotational axis. However, due to high frequency variations of vehicle in practice, the actual rotational axis, which is denoted as *x⃑*^(*n*)^, always offsets from its assuming observation, thus an error model is constructed for *x⃑*^(*n*)^ as follows:

(17) $${\vec{x}}^{\left(n\right)}={\left[\sin\zeta \sin\eta \;\sin\zeta \cos\eta \;-\cos\zeta \right]}^{T}$$

where *ζ* represents the angle between *x⃑*^(*n*)^ and *x⃑*^(*n*)^′, and it follows a normal probability distribution. *η* is the orientation of the projection vector of *x⃑*^(*n*)^ with respect to the true north, and it follows a uniform probability distribution in the interval of [0,2*π*]. Using *ζ* and *η* defined above, the actual rotational axis of the land vehicle can be modeled in a rather simple manner.

In short-baseline cases, the dominant errors of DD carrier phase measurements include multipath, which can be considered noise-like, and receiver thermal noise [19]. Determine each ∇Δ*N^i^* with the true ambiguity resolution, then ${\tilde{\vec{a}}}^{\left(n\right)}(t_{k})'$ and *a⃑*^(*n*)^(*t_k_*)′ can be obtained from Equations (3) and (4), respectively. By a minus operation, we have:

(18) $$\begin{array}{l} {\underline{\tilde{\vec{a}}}}^{\left(n\right)}(t_{k})'={\underline{\vec{a}}}^{\left(n\right)}(t_{k})'+\delta {\underline{\vec{a}}}^{\left(n\right)}(t_{k})'\\ \delta {\underset{⇀}{\vec{a}}}^{\left(n\right)}(t_{k})'=\delta {\vec{a}}^{\left(n\right)}(t_{k})'-\left({\tilde{\vec{a}}}^{\left(n\right)}(t_{k}){'}^{T}\cdot \delta {\vec{x}}^{\left(n\right)}\right){\vec{x}}^{(n)'}\\ -\left({\tilde{\vec{a}}}^{\left(n\right)}(t_{k}){'}^{T}\cdot {\vec{x}}^{\left(n\right)}\right)\delta {\vec{x}}^{\left(n\right)}-\left(\delta {\vec{a}}^{\left(n\right)}{\left(t_{k}\right)}^{'T}\cdot {\vec{x}}^{\left(n\right)}\right){\vec{x}}^{\left(n\right)} \end{array}$$

where *δa⃑*^(*n*)^(*t_k_*)′ denotes the stochastic error vector deviating from the true baseline vector resolution. The variance covariance matrix of ∇Δ*b⃑_ϕ_* is given by *W*_∇Δ*b⃑*_ . Hence, the errors in *x⃑*^(*n*)^′ and ${\tilde{\vec{a}}}^{\left(n\right)}(t_{k})'$ can induce composite effect on the testing objective. It can be explained by the formula as follows:

(19) $$\begin{array}{l} {\underline{\tilde{\vec{a}}}}^{\left(n\right)}(t_{k})'={\underline{\vec{a}}}^{\left(n\right)}(t_{k})'+\delta {\underline{\vec{a}}}^{\left(n\right)}(t_{k})'\\ \delta {\underline{\vec{a}}}^{\left(n\right)}(t_{k})'=\delta {\vec{a}}^{\left(n\right)}(t_{k})'-\left({\tilde{\vec{\text{a}}}}^{\left(n\right)}{{(t}_{k})}^{'T}\cdot \delta {\vec{x}}^{\left(n\right)}\right){\vec{x}}^{(n)'}\\ -\left({\tilde{\vec{a}}}^{\left(n\right)}{\left(t_{k}\right)}^{'T}\cdot {\vec{x}}^{\left(n\right)}\right)\delta {\vec{x}}^{\left(n\right)}-\left(\delta {\vec{a}}^{\left(n\right)}{\left(t_{k}\right)}^{'T}\cdot {\vec{x}}^{\left(n\right)}\right){\vec{x}}^{\left(n\right)}. \end{array}$$

Equation (19) is also true to each ambiguity candidate in the search space. With Equations (14) and (19), both *δθ* and *δa⃑*^(*n*)^(*t_k_*)′ can expand the misleading impact of the error-included testing objective. It implies that the true ambiguity combination ∇Δ*N⃑* may be filtered out. Thus the threshold value should be chosen appropriately to make sure that ∇Δ*N⃑* is always kept in the search space, and the search space is shrinking constantly by the rate-gyro-integral constraint imposed.

## 5. Simulations

Basing on the implementation method and the models constructed for various measurement errors, some simulations are conducted. For simplicity, the measurement errors of IMU and GNSS receivers, plus the actual rotational axis offsets are addressed as the 1st, 2nd and 3rd type measurement error, respectively. For different simulation scenarios, the results were assessed on two aspects, *i.e.*, the success rate and the shrinking efficiency. The simulation experiments are carried out by means of the basic steps as follows:

Step 1 *a⃑*^(*b*)^ = [3 0 0]*^T^* and $C_{b}^{n}(t_{o})=I$ are chosen, respectively, and the updating frequency of GNSS measurements is chosen as 1 Hz;
Step 2 with the actual locations and GPS constellation imposed, select a reference satellite and three master satellites, then compute *G_i_* with Equation (3);
Step 3 set the parameters for rotation axis *x⃑*^(*n*)^, the complete rotational angle *θ*, and angle velocity vector ${\vec{\omega }}_{nb}^{b}(\tau )={\left[0\;0\;\omega (\tau )\right]}^{T}$, respectively, then compute *a⃑*^(*n*)^ (*t_k_*) from:
  (20) $${\vec{a}}^{\left(n\right)}(t_{k})=\left\{I-\sin\theta \left[W^{{\vec{x}}^{\left(n\right)}}\right]+(1-\cos\theta )\cdot {\left[W^{{\vec{x}}^{\left(n\right)}}\right]}^{2}\right\}{\vec{a}}^{\left(n\right)}(t_{0}).$$
  with [13]:
  (21) $$\begin{array}{cc} W^{{\vec{x}}^{\left(n\right)}}= & \left[\begin{array}{ccc} 0 & n_{3} & -n_{2}\\ -n_{3} & 0 & n_{1}\\ n_{2} & -n_{1} & 0 \end{array}\right]\\ {\vec{x}}^{\left(n\right)}= & {\left[n_{1}\;n_{2}\;n_{3}\right]}^{T},\;\theta =\left|\int_{t_{0}}^{t_{k}}{\vec{\omega }}_{nb}^{b}(\tau )d\tau \right|. \end{array}$$
Step 4 with the selected satellites in Step 2 imposed, generate the true ambiguity vector ∇Δ*N⃑* (3-D) and a set of DD carrier phase observations for each *t_k_*, then construct an initial ambiguity search space □ whose center is fixed at ∇Δ*N⃑* and the search radius is defined as a random variable with a standard deviation of 5 cycles;
Step 5 for each candidate lies inside ℤ at *t_k_*, compute the corresponding testing objective Δ*α_k,m_*;
Step 6 test all the Δ*a*_*k*,*m*_ s for each *t_k_*, filter out the candidates which satisfy |Δ*α_k,m_*| > |Δ*α*|_threshold_ from ℤ.

In Step 2, the actual GNSS data was collected on 11 June 2011, at N 29.5650°, E 106.2197°. From the satellites in view, the satellite with maximal elevation is chosen as the reference satellite, then three master satellites are selected based on the minimal GDOP principle. In Step 4, the parameter of search radius, *i.e.*, 5 cycles, is decided by “current” average accuracy level of DD code measurement [19].

In the first simulation experiment, except for the 1st type measurement error, both the 2nd and 3rd type measurement errors are considered. *ζ* has a normal distribution *N*(0, 3°2) and the standard deviation associated with DD carrier phase is 0.05 cycle. If ${\vec{\omega }}_{nb}^{b}(\tau )={\left[0\;0\;10{}^{\circ}/s\right]}^{T}$ and 1 Hz GNSS update frequency are chosen, total 18 GNSS updates will be generated for a 180° rotation procedure. Five different thresholds, *i.e.*, 0.1°, 0.5°, 1°, 3° and 5°, are selected. For each possible combination of thresholds and error types, the simulation is repeated 10,000 times. The success rate is defined as the percentage of occurrences that the steady search space contains the true ambiguity combination. Table 1 shows the success rates vary with function of the threshold values.

**Figure 3.**
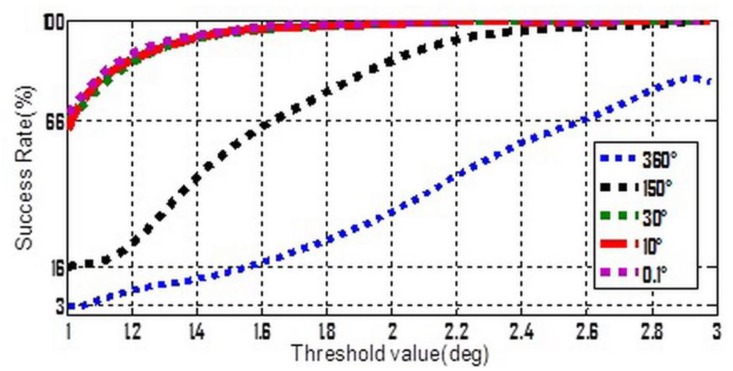
Success rate varies with threshold at different accuracy levels of gyroscope.

**Figure 4.**
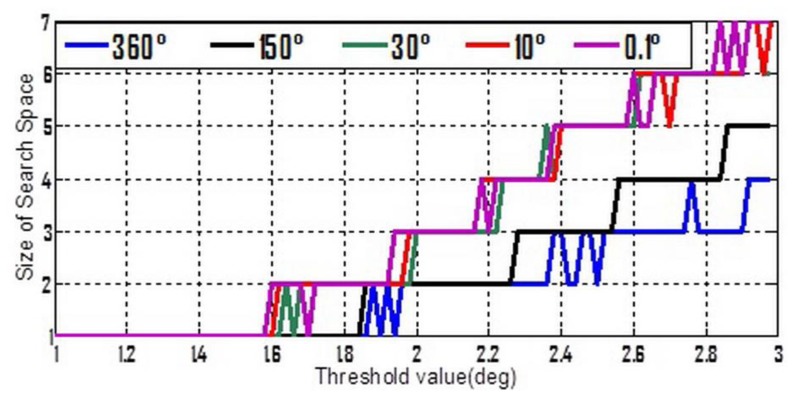
Shrinking efficiency varies with threshold at different accuracy of gyroscope.

In the second one, all the three types of measurement errors are all taken into account. The simulation parameters for Equation (13) are selected in accordance with “current” accuracy levels of gyroscopes [18], which are given in Table 2:

To make Equation (13) simpler, three stochastic constant biases, whose mean value and standard deviation are both consistent with each other, and consist of the three components of $\delta {\vec{\omega }}_{ib}^{b}$. The average value is zero but five different standard deviation values ranging from 0.1° to 360° were chosen. Moreover, from Table 1 it can be seen that whatever the 2nd or 3rd type error is considered, the success rates seem to be acceptable if the threshold value lies in the interval of [1°,3°]. So [1°,3°] was divided into 100 equal parts, and a set of threshold values can be formed by the separate points. Keep the other settings unchanged, the simulation is repeated 10,000 times for each possible combination of standard-deviation and threshold value. The success rates and shrinking efficiency are shown in Figures 3 and 4, respectively.

Shrinking efficiency herein is explained by the size of steady ambiguity search space, which is concluded by averaging and rounding figures obtained from a lot of simulations for the successful shrinking procedures.

In Figure 3, if the accuracy of gyroscope is high enough, e.g., a tactical grade IMU, the measurement errors of gyroscope will no longer have a dominant effect on the success rate. In this case, the 2nd and 3rd types of measurement errors are believed to be much more influential. Figure 4 shows that a relative lower threshold can promote the shrinking efficiency. In addition, an interesting tendency can be seen from Figure 4. For a fixed threshold value, a relative higher accuracy of gyroscope can reduce the shrinking efficiency (larger size of the steady search space). This tendency will become even evident if the threshold value is higher than 1.6°.

## 6. Land Vehicle Testing

**Figure 5.**
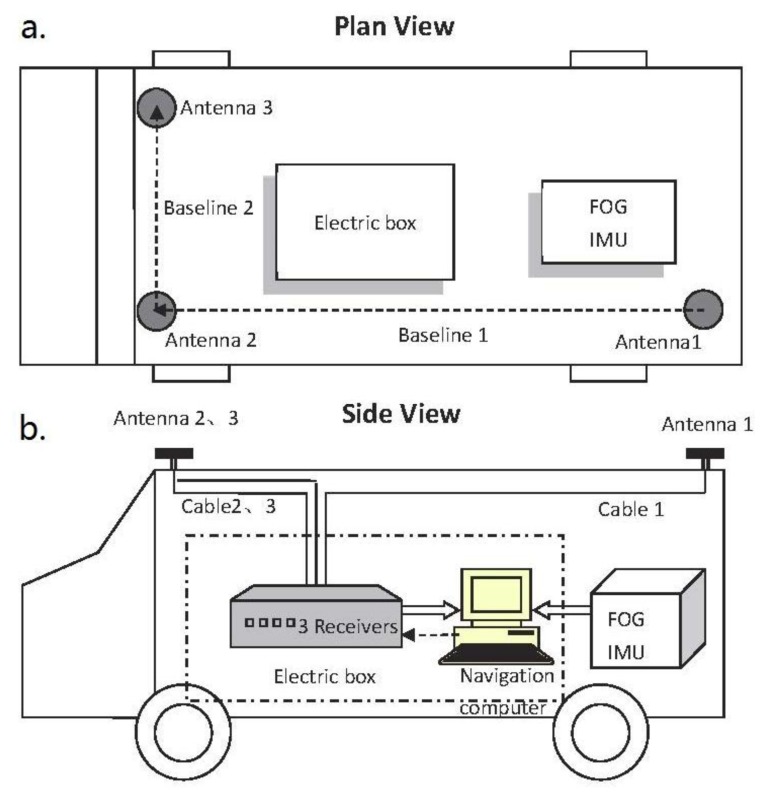
Testing vehicle is set up: (**a**) plan view; (**b**) side view.

The GNSS/INS integrated attitude determination system used herein primarily consists of a tactic grade FOG-IMU, an array of three GNSS antennas with receivers connected individually and a navigation computer. The concerned technical characteristic of the FOG-IMU is the equivalent bias of gyroscope denoted as *b* , which satisfies *b* < 4°/h in normal temperature circumstances. The Novatel GPS-701 antenna features a steady electrical phase center. The type of receivers is Novatel OEMV-1G, with C/A code measurement precision of 6 cm RMS and the carrier phase measurement precision of 0.75 mm RMS. Three antennas are approximately arranged in a right triangle pattern with 4.634 m baseline1 and 1.544 m baseline2, as shown in Figure 5(a).

**Figure 6.**
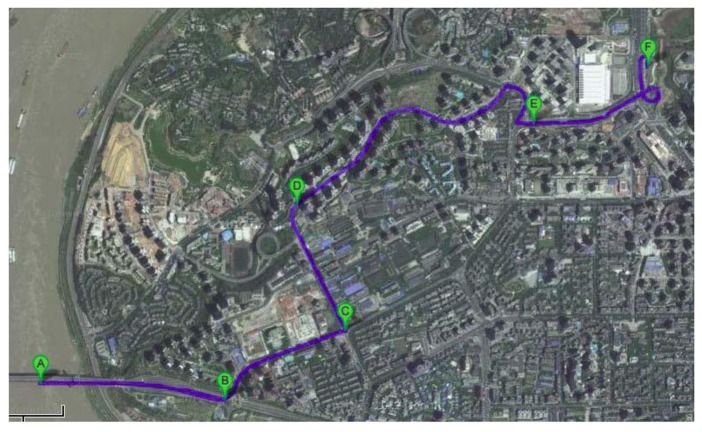
Testing trajectory.

The actual field data was collected on 16 April 2008 in the Chong Qing urban area, China. The GPS measurements were available at a rate of 1 Hz and the output rate of IMU was 200 Hz. After a successful static initialization, the vehicle was driven into the dense urban area where it followed the trajectory shown in Figure 6 for about 5 min. It can be seen that there were five evident curves on the test route. These curves were denoted as B, C, D, E and F sequentially. Then five data sections were extracted from these curves, and Sections B and D were abandoned due to quite poor GPS satellite visibility.

**Figure 7.**
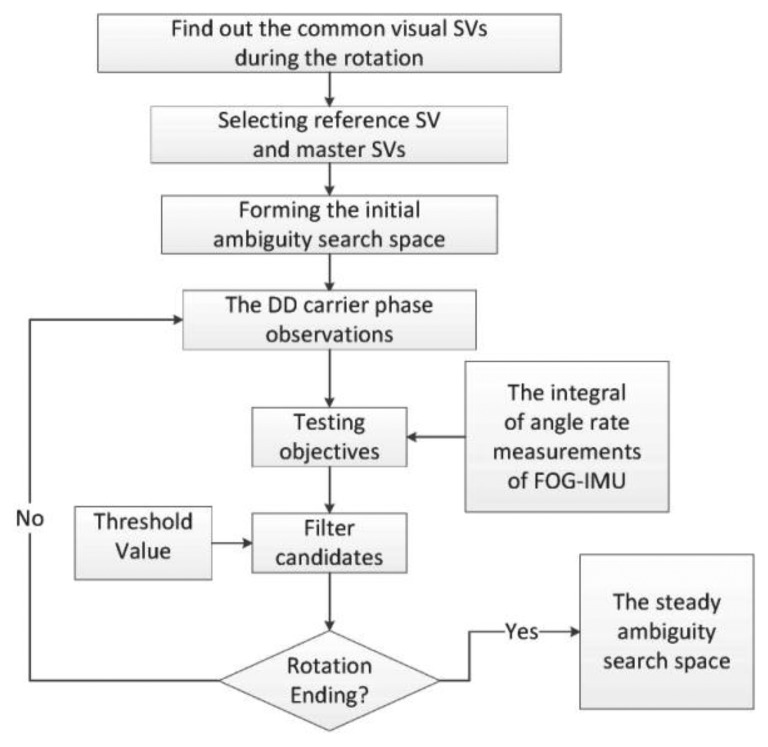
Scheme for actual field data processing.

Each data section selected is processed following the scheme shown in Figure 7. This scheme is essentially coincident with the simulation steps mentioned in the former section, and it is required that at least four satellites should be tracked uninterruptedly by all the three receivers during collecting the data section.

The initial ambiguity search space (3D) is determined by an float ambiguity estimation vector denoted as $\nabla \Delta \hat{\vec{N}}=\left\{\nabla \Delta {\hat{N}}_{1}\nabla \Delta {\hat{N}}_{2}\nabla \Delta {\hat{N}}_{3}\right\}$, whose components are computed by using the DD code and carrier phase measurements, and a search radius of five cycles is selected. Thus the initial search space □ contains 1,331 candidates. As the measurement precision of Novatel OEMV-1G is 6 cm RMS in terms of C/A code, a “5 cycles” search radius is adequate. To judge that whether the shrinking procedure of the search space □ is successful or not, the true ambiguity combination is obtained in advance by means of backward processing for the integrated navigation attitude results and associated measurements.

### 6.1. Feasibility Test

**Figure 8.**
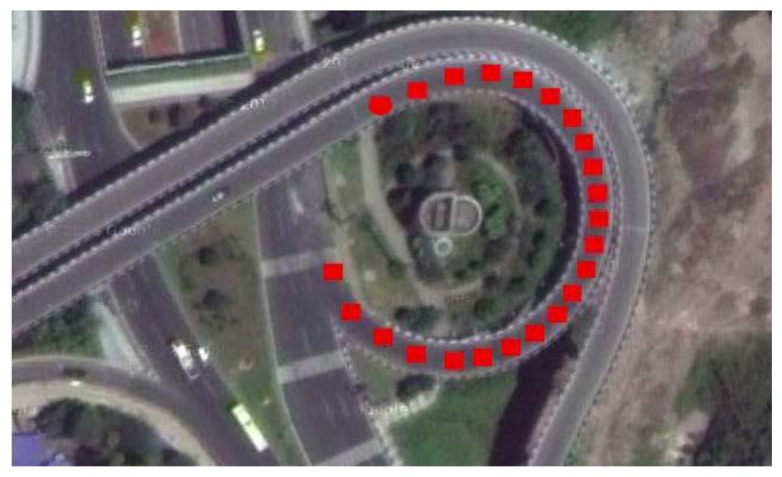
Turntable road and locations of 23 GPS epochs for data section F.

**Figure 9.**
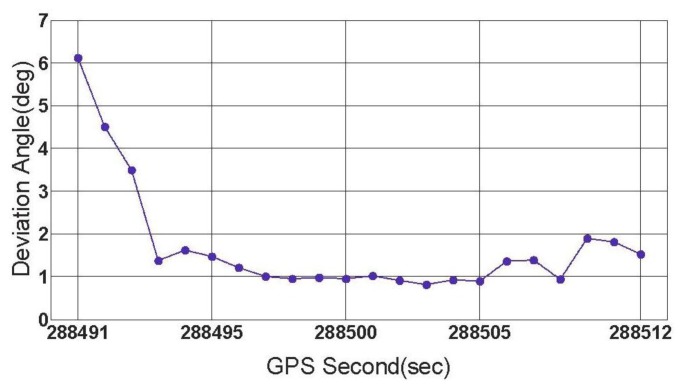
ζ s for data section F.

**Figure 10.**
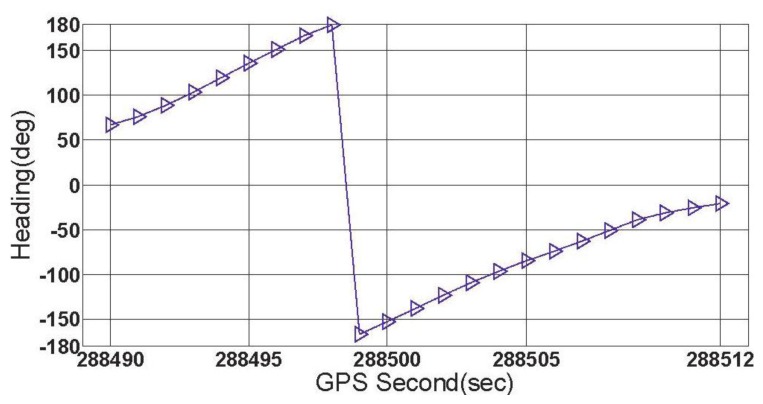
Vehicle orientations for data section F.

In this subsection, the feasibility of the rate-gyro-integral constraint in actual applications is tested with data section C, E and F. During the collecting period of data section F, the vehicle was driven along a turntable road shown in Figure 8. As can be noted, the ends of this section were respectively connected with a viaduct and an underground passage of the viaduct. Hence, over this region, the vehicle featured tilt attitude (roll and pitch) with the level of several degrees, see Figure 9. The estimation method for *ζ* comes from [12]. Data section F includes a total of 23 GPS epochs, which are expressed on the turntable road by red blocks in Figure 8. From Figure 10, it can be seen that the orientations of vehicle, which were provided by the integrated attitude determination system, varied continuously clockwise. Moreover, totally 6 GPS satellites were tracked continuously by all the three receivers over this region.

**Figure 11.**
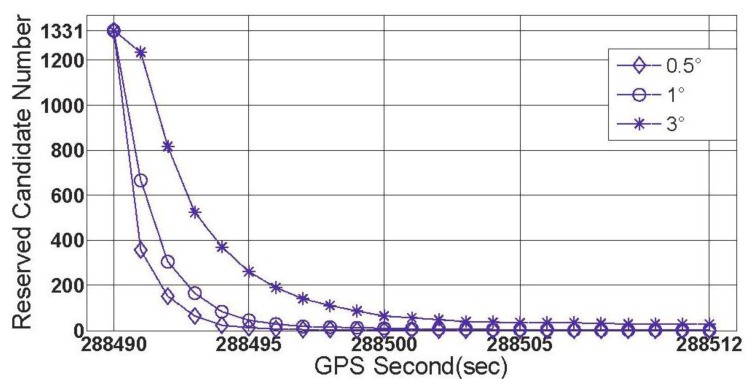
The first group of results of feasibility test (data section F).

When the threshold value is chosen to be 0.5°, 1° and 3 °, respectively, the first group of results of feasibility test can be obtained by processing data section F and given by Figure 11.

Figure 11 shows three successful shrinking procedures of □ . The size of steady search space are 2 and 30 for the cases of |Δ*α*|_threshold_=1° and |Δ*α*|_threshold_=3°,respectively. If |Δ*α*|_threshold_=0.5° is chosen, for data section F, the true ambiguity combination will be locked only by using the rate-gyro-integral constraint.

**Figure 12.**
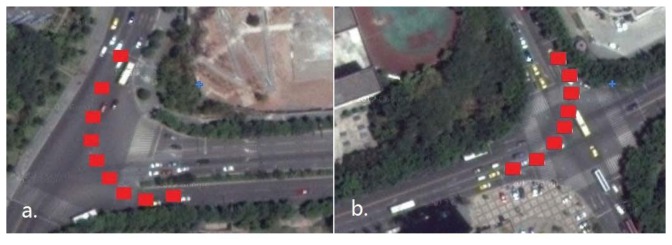
(**a**) T-junction and locations of 9 GPS epochs; (**b**) Crossroad and locations of 8 GPS epochs.

**Figure 13.**
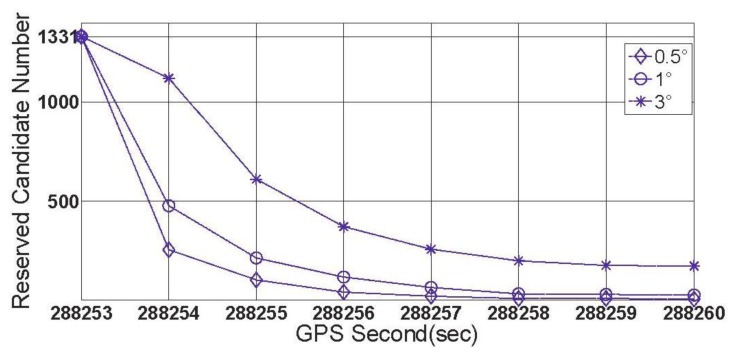
The second group of the results for the feasibility test (data section C).

**Figure 14.**
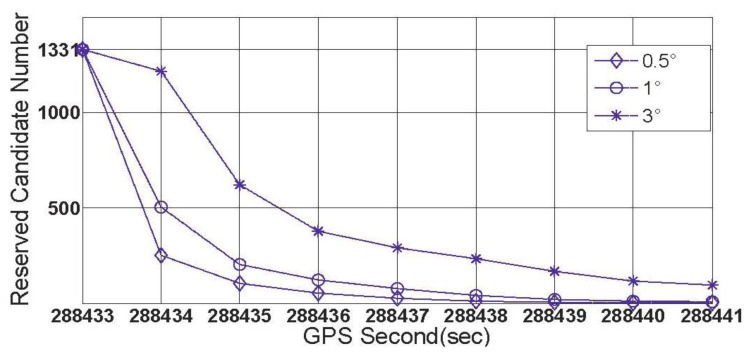
The third group of the results for the feasibility test (data section E).

Data section C was collected on a crossroad (Figure 12(b)) with eight GPS epochs included and seven common visual satellites tracked. The vehicle turned at about 80° counter-clockwise over this region. Data section E was collected on a T-junction (Figure 12(a)) with the turning angle reaching up to 100°, and the numbers of GPS epochs and common visual satellites were 9 and 6, respectively. Each of the two curves has a turning angle much less than that of data section F, but they are more common than turntables in urban. The locations of GPS epochs for both data section C and E are noted by red blocks in Figure 12(a,b). With the data sections C and E processed, the second and third groups of the results for the feasibility test are given by Figures 13 and 14, respectively.

From Figures 13 and 14, it is known that for the crossroads and T-junctions, the most common types of curves in urban areas, both the success rate and shrinking efficiency can be guaranteed by adequate common visual satellites. Although the true ambiguity combination cannot be fixed in either C or E case, the method of rate-gyro-integral constraint is shown to be an efficient way of shrinking the size of the search space ℤ to acceptable levels in practice.

The three groups of results demonstrate that if adequate common visual satellites are available, as well as the turning angle is large enough, the rate-gyro-integral constraint is practicable in land navigation applications.

### 6.2. Characteristics Test

**Figure 15.**
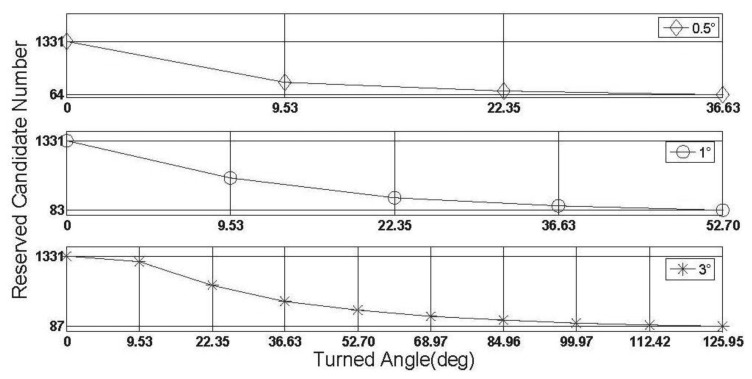
Size of ℤ contracts to less than 100 for each |Δ*α*|_threshold_.

**Figure 16.**
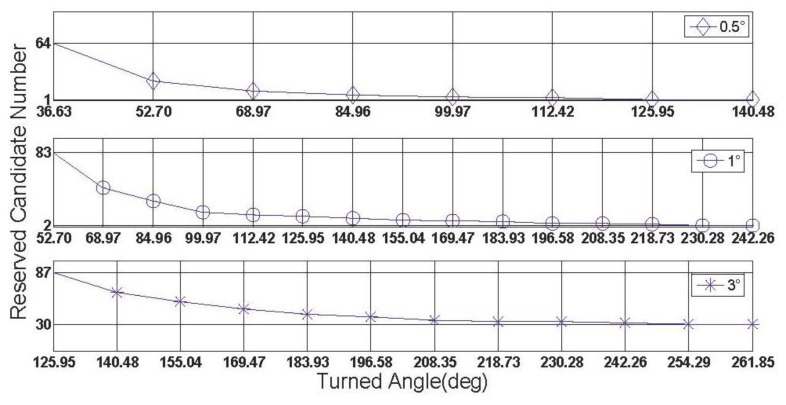
Size of the search space □ obtains a steady status for each |Δ*α*|_threshold_.

In this subsection, some characteristics of the rate-gyro-integral constraint will be carried out by use of data section F. It is not difficult to know that in those successful shrinking procedures, lower threshold value can promote the shrinking efficiency. To verify this characteristic in practical cases, two zooms to Figure 11 are presented in Figures 15 and 16.

**Figure 17.**
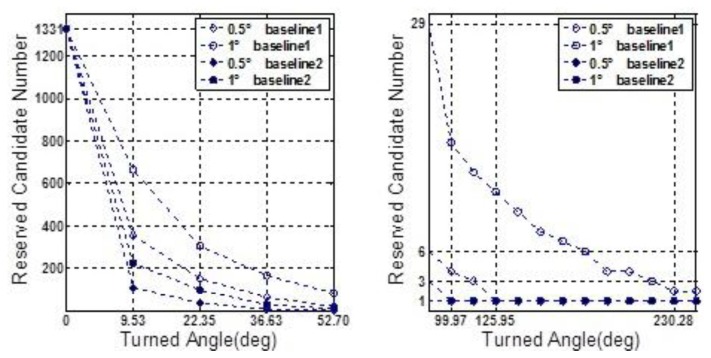
Comparison of shrinking efficiencies of Baselines 1 and 2.

In both Figures 15 and 16, it is noted that the turning angle of vehicle at each epoch is used as the argument, instead of the GPS second in Figure 11. According to Equation (11), a major contributor to weakening the performance of rate-gyro-integral constraint is increasing the length of baseline. By now, only baseline1 has been considered in processing actual field data sections. By use of the same processing scheme and data sections, the results of baseline2 are presented here for a comparison purpose. Figure 17 shows the shrinking processes of the search space □ when both Baseline1 and Baseline2 were considered.

After each data element with turning angle larger than 190° was excluded from data section F, an approximate 1/2 cycle turning is forced. From this preserved data section, four subsequences will be picked out and each of them has a relative even distribution on turning angle. Table 3 lists four subsequences under the condition of |Δ*α*|_threshold_=1° and approximate 1/2 cycle turning size of the search space □ .

From Table 3, it is noted that all the subsequences have common elements when the turning angles are 85° and 183.9°, which are relatively close to 90° and 180°, respectively. Hence, they are referred as “1/4 cycle” and “1/2 cycle” positions. Overall, for each turning angle, the search spaces □ from different subsequences are on the same level of size, especially at the “1/2 cycle” position. But for the “1/4 cycle” position, the search space □ from the “3 points” subsequence has a relatively larger size than the others. Hence, for the rotation procedure, it is suggested that the intervals of adjacent GNSS updates should be less than 45°. But with the intervals, which are less than 45°, becoming smaller, the performance of the rate-gyro-integral constraint can thus be only slightly improved.

## 7. Conclusions

In this paper, a new constraint for use in GNSS attitude determination in land vehicle applications has been developed and analyzed. To speed up the initialization of attitude parameters under dynamic rotation circumstances, the new method of applying a constraint to vehicle turning motion has been assessed. It has been demonstrated that this new constraint can result in high ambiguity search success rates and efficiency for shrinking the ambiguity search space. The contributing factors to the actual performance of the new rate-gyro-integral constraint can be divided into two categories, namely the systematical factors and the measurement factors respectively. The systematical factors, which contain the length of baseline and the amplitude of turning angle, determine the attained peak value of the testing objective, while the accuracy of computed testing objective depends on the measurement factors, which include the IMU measurement errors, especially those associated with angle rate, GNSS carrier phase measurement errors and the actual turning axis offsets. Additionally, the threshold has an important effect on the performance of the new constraint as well.

The proposed constraint has been verified with simulations as well as actual field data. In the simulations, both the successful rate and shrinking efficiency have been analyzed in different measurement scenarios, especially with different grades of inertial sensors considered. Our vehicle test, with the data collected in the urban area of Chong Qing, covers three typical curves in urban areas. From the obtained results, we can conclude that the rate-gyro-integral constraint is practical in land navigation applications. Although only using a tactic grade IMU in our vehicle test, it is emphasized that the new constraint is generally applicable and thus also applicable to the GNSS/INS integrated system by use of low grade MIMU.

The idea behind the proposed new rate-gyro-integral constraint for GNSS attitude determination is the utilization of the platform dynamic information measured by affordable inertial sensors. Hence, further research may focus on the use of the rate-gyro-integral constraint to enhance the strength of GNSS model under the poor observation circumstances which are very common for the operations of dynamic platforms.

## Figures and Tables

**Table 1.** Success rates with the 2nd or 3rd type measurement error considered individually.

	**5°**	**3°**	**1°**	**0.5°**	**0.1°**
2nd Type	100%	100%	82.46%	5.19%	0%
3rd Type	100%	99.98%	95.05%	68.18%	1.46%

**Table 2.** “Current” accuracy levels of gyroscopes.

	**b (°/h)**	**w(°/√h)**
Middle grade	0.1	<0.03
Tactic grade	1∼100	0.03∼0.1
Automotive grade	>100	>1

**Table 3.** Shrinking processes of the search space □ from different subsequences.

	**0°**	**9.5°**	**22.4°**	**36.6°**	**52.7°**	**69°**	**85°**
14p	1331	665	306	166	83	44	29
7p	1331	–	306	–	88	–	30
5p	1331	–	–	179	–	–	31
3p	1331	–	–	–	–	–	79
	100°	112.4°	126°	140.5°	155°	169.5°	183.9°
14p	17	14	12	10	8	7	6
7p	–	14	–	–	8	–	6
5p	–	–	–	11	–	–	7
3p	–	–	–	–	–	–	7

## Appendix

If both *α*_0_ ∈ [0, 2*π*] and $\theta \in [0,2\pi -\sin^{-1}\left(\frac{\delta L\sin\alpha_{0}}{L}\right))$ are satisfied, it is obvious that:

(A.1) αk,m'=tan2−1(Lsinθ+δLsinα0Lcosθ+δLcosα0)−tan2−1(δLsinα0L+δLcosα0).

However, it should be note that once *θ* reaches up to $2\pi -\sin^{-1}\left(\frac{\delta L\sin\alpha_{0}}{L}\right)$, *i.e.*, the denominator in the brackets of the first term in Equation (A.1) is equal to zero, we will obtain that:

(A.2) αk,m'=−tan2−1(δLsinα0L+δLcosα0)∉[0,2π].

This value cannot reflect the actual rotational angle between *a⃑*^(*n*)^ (*t*_0_)′*_m_* and *a⃑*^(*n*)^ (*t_k_*)′*_m_* . In the case of $\theta \in [2\pi -\sin^{-1}\left(\frac{\delta L\sin\alpha_{0}}{L}\right),2\pi ]$, hence, the analytic formula of *α*_*k*,*m*_′ should be given by:

(A.3) αk,m'=tan2−1(Lsinθ+δLsinα0Lcosθ+δLcosα0)−tan2−1(δLsinα0L+δLcosα0)+2π.

Finally, together with Δ*α_k,m_* =*α_k,m_*^′^ −*θ*,the derivation of Equation (7) is completed.
